# A case of locally advanced adenosquamous carcinoma of the cecum with long-term survival

**DOI:** 10.20407/fmj.2019-007

**Published:** 2019-09-25

**Authors:** Hiroshi Matsuoka, Mitsuru Nakagawa, Tsunekazu Hanai, Koji Masumori, Yoshikazu Koide, Hidetoshi Katsuno, Tomoyoshi Endo, Masahiro Mizuno, Yongchol Chong, Koutarou Maeda, Testuya Tsukamoto, Ichiro Uyama

**Affiliations:** 1 Departments of Surgery, Fujita Health University, School of Medicine, Toyoake, Aichi, Japan; 2 Department of Pathology, Fujita Health University, School of Medicine, Toyoake, Aichi, Japan; 3 International Medical Center, Fujita Health University Hospital, Toyoake, Aichi, Japan

**Keywords:** Adenosquamous carcinoma, Colorectal cancer, Long-term survival

## Abstract

A 63-year-old woman was admitted to our hospital with a right lower abdominal mass and general fatigue. Preoperative examination suggested a large ovarian tumor or cecal carcinoma. However, her intraoperative diagnosis was colon cancer; we therefore performed an ileocecal resection with oophorectomy. The tumor was pathologically diagnosed as adenosquamous carcinoma T4bN1M-stage IIIa. We administrated CapeOX adjuvant chemotherapy for 6 months. Adenosquamous carcinoma is extremely rare, at around 0.1% of all colorectal cancers, and usually has a poor prognosis. The patient is still alive without recurrence after 84 post-operative months, even with later developments of metachronous early colorectal cancer and breast cancer. We herein report a rare case of cecal ASC with good prognosis.

## Introduction

More than 90% of colorectal cancer (CRC) is well-to-moderately differentiated adenocarcinoma. Primary adenosquamous carcinoma (ASC) of the colon is extremely rare. It has high malignant potential and its long-term prognosis is usually unfavorable.^1^ Here, we report a rare case of cecal ASC with good prognosis.

## Case report

A 63-year-old woman was admitted to our hospital with a right lower abdominal mass. She had no relevant previous medical or family history. On admission, her height and body weight were 145 cm and 40 kg, respectively. Her initial laboratory data showed severe anemia, with hemoglobin levels of 5.6 g/dL, red blood cell count of 3.888×10^6^/μL, and platelet count of 69.4×10^4^/μL, as well as slight transaminitis and proteinemia. Marker antigen levels were carcinoembryonic antigen (CEA): 1.9 ng (normal: <5.0); carbohydrate antigen 19-9 (CA19-9): 0.1 U/mL (normal: <37); and squamous cell carcinoma (SCC) antigen: 0.6 ng/mL (normal: <1.5).

**Figure 1:**
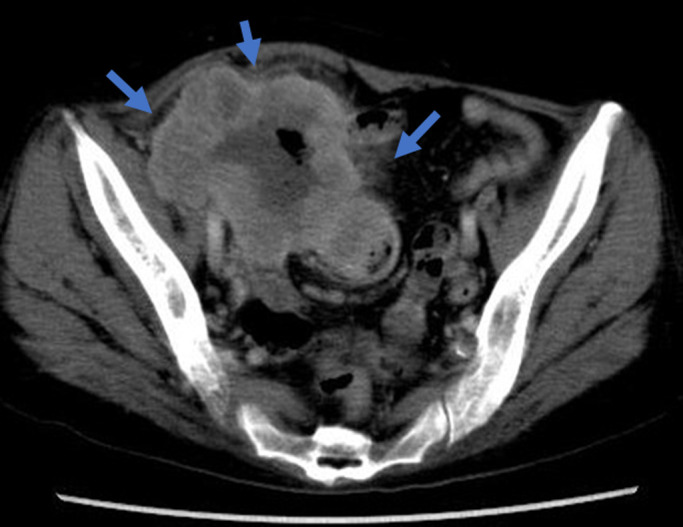
Abdominopelvic contrast-enhanced computed tomography shows a large mass of the cecum and right ovary with well-contrasted circumference vessels.

**Figure 2:**
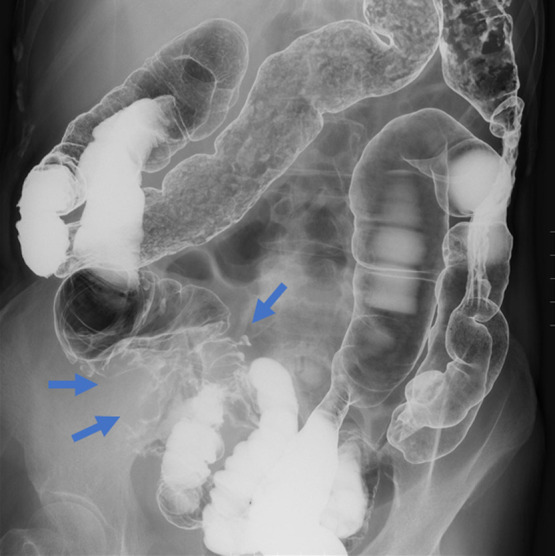
Cecum wall thickness and obstruction showing severe “apple core sign.”

Abdominopelvic contrast-enhanced computed tomography (CT) showed a large mass involving the cecum and the right ovary, with well-contrasted circumference vessels. The tumor size was 100×80×70 mm, and was of suspected cecal origin, with swelling of the mesenteric lymph nodes (Figure 1). Barium enema findings showed cecal wall thickness and obstruction with a “apple core sign”; however, barium flow to the ileum was good (Figure 2).

Colonoscopy and biopsy could not be performed as the patient refused; therefore, histological diagnosis was not possible. Surgery was performed with a pre-operative diagnosis of carcinoma with the suspected origin in cecum or right ovary. During surgery, we identified the origin of this tumor as a cecal carcinoma, with invasion to the retroperitoneum and right ovary, as well as the right external iliac artery. We performed an ileocecal resection with bilateral oophorectomy, partial resection of iliopsoas muscle, and D3 lymph node resection. We observed no evidence of peritoneum dissemination or hepatic metastases.

**Figure 3:**
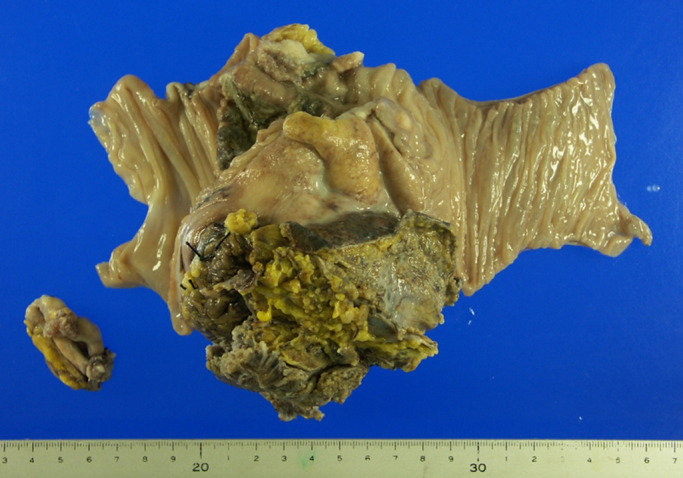
Ileocecum specimen shows type 2 cancer in the cecum.

**Figure 4:**
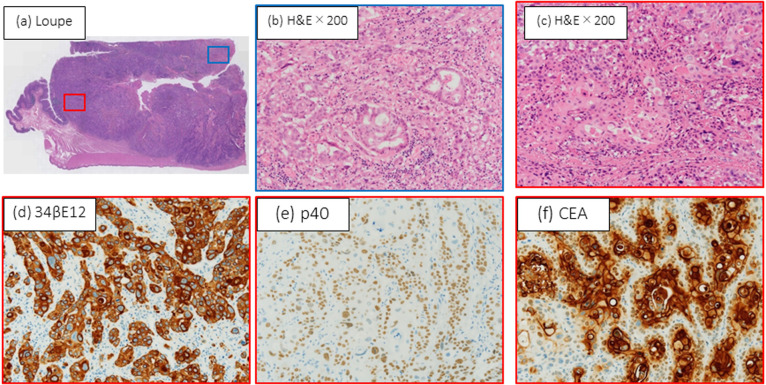
Microscopic and immunohistochemical features of adenosquamous carcinoma of the cecum. (a) Histological findings. Blue: adenocarcinoma (AD) component; red: squamous cell carcinoma (SCC) component (hematoxylin and eosin [H&E]; loupe). (b) Ductal and cribriform pattern of atypical columnar AD cells (H&E; 200× magnification). (c) Island pattern by dysplastic squamous epithelium with keratinization in SCC component (H&E; 200× magnification). Immunohistochemistry shows SCC component is (d) strongly 34βE12+, (e) p40+, and (f) focally positive for carcinoembryonic antigen ( 200× magnification for each).

Pathological findings showed a type 2 tumor, measuring 10 cm in diameter at the cecum, that had invaded to the right ovary and iliopsoas muscle. The tumor was pathologically diagnosed as adenosquamous cell carcinoma, T4b (retroperitoneum), int, INFβ, ly2, v1, n1(2/11), *2RAS*-wild type. Immunohistochemical examination showed it to be MLH1+, PMS2+, and MSH6+ (Figures 3, 4).^2^

The patient had no postoperative complications. She received eight courses of adjuvant chemotherapy with CapeOX (capecitabine 2000 mg/m^2^ PO d1–14, oxaliplatin 130 mg/m^2^ civ d1) over 6 months. A colonoscopy performed 18 months after the initial surgery found an Isp polyp at the sigmoid colon, for which an endoscopic mucosal resection was performed. Pathological findings of the polyp revealed a well-differentiated adenocarcinoma, depth 500 μm, ly0, v0. A follow-up CT 7 years after the initial surgery revealed a tumor of the right breast, diagnosed as invasive ductal carcinoma, and resected. The patient is currently receiving postoperative adjuvant hormonal therapy and is alive 8 years after the first surgery, with no recurrence of colonic ASC.

## Discussion

Japanese clinical guidelines for colon cancer define ASC of the colon as a neoplasm that comprises adenocarcinoma and SCC. The histogenesis of ASC of the colon is not clearly understood, but three hypotheses have been suggested: (1) undifferentiated or reverse cells in the colonic epithelium may change directly into SCC; (2) secondary ASC derived from adenocarcinoma (currently, the most widely accepted explanation); and (3) ectopic squamous cells in the colonic mucosa may be directly transformed into squamous malignant cells.^3,4^ ASC of the colon is rare, with an incidence of 0.1%−0.2% among all CRCs.^5^ Masoomi et al. reported a population-based evaluation of ASC of the colon in the California Cancer Registry from 1994 to 2004; they found that out of 111,263 cases of CRC, ASC was identified in only 99 cases (0.09%).^6^ The most common location was the proximal colon and the median age was 67 years, which is similar to our present case. As colorectal ASC tends to form both regional and distant metastases, its long-term prognosis is often unfavorable, with an overall 5-year survival rate of only 31%. A study of 576 patients with ASC in Taiwan showed 27 had colorectal ASC (0.04%); it found that colorectal ASC was most likely to affect the respiratory system (73.8%), followed by the alimentary canal (16.2%), and the female reproductive tract (10%), and that prognosis for ASC of the colon was much worse than for other primary ASCs.^7,8^ Data from the National Institutes of Health also showed a poor prognosis, which Hijikawa et al. suggested was due to the very rapid growth of secondary ASC derived from adenocarcinoma.^4^ Our present patient had no distant metastases, but as she had lymph node metastases and T4b tumor depth, she had a high risk of recurrence. She received adjuvant chemotherapy using CapeOX for colon adenocarcinoma; however, the exact role of adjuvant chemotherapy remains unclear.^9^

In the present case, the patient developed early sigmoid colon cancer at 1 year after the first surgery and advanced breast cancer at 7 years after. Metachronous colon cancer is a criterion for Lynch syndrome in the Revised Bethesda guidelines. Raymond et al. reported the possibility of Lynch syndrome-associated breast and prostate cancers.^10^ In the present case, the specimen was immunopositive for MLH1, PMS2, and MSH6; however, these proteins are not clearly described in the criteria and we conclude this case is not Lynch syndrome. Recently, Duncan et al. reported high microsatellite instability in ASC and ASC with Lynch syndrome.^11–13^

As no biomarkers for diagnosis and prognosis are currently known, we should treat ASC as conventional adenocarcinoma. In the future, we expect genetic analysis using next-generation sequencing to assist in drug and treatment choice. Erik et al. reported molecular profiling of lung ASC.^14^ Because of the rarity of colonic ASC, countrywide registry database systems and gene analysis programs are required.
